# Compound Fault Diagnosis of a Wind Turbine Gearbox Based on MOMEDA and Parallel Parameter Optimized Resonant Sparse Decomposition

**DOI:** 10.3390/s22208017

**Published:** 2022-10-20

**Authors:** Yang Feng, Xiangfeng Zhang, Hong Jiang, Jun Li

**Affiliations:** College of Mechanical Engineering, Xinjiang University, Urumqi 830017, China

**Keywords:** compound fault diagnosis, feature extraction, gearbox, sparse representation

## Abstract

Wind turbines usually operate in harsh environments. The gearbox, the key component of the transmission chain in wind turbines, can easily be affected by multiple factors during the operation process and develop compound faults. Different types of faults can occur, coupled with each other and staggered interference. Thus, a challenge is to extract the fault characteristics from the composite fault signal to improve the reliability and the accuracy of compound fault diagnosis. To address the above problems, we propose a compound fault diagnosis method for wind turbine gearboxes based on multipoint optimal minimum entropy deconvolution adjusted (MOMEDA) and parallel parameter optimized resonant sparse decomposition (RSSD). Firstly, the MOMEDA is applied to the preprocess, setting the deconvolution period with different fault frequency types to eliminate the interference of the transmission path and environmental noise, while decoupling and separating the different types of single faults. Then, the RSSD method with parallel parameter optimization is applied for decomposing the preprocessed signal to obtain the low resonance components, further suppressing the interference components and enhancing the periodic fault characteristics. Finally, envelope demodulation of the enhanced signal is applied to extract the fault features and identify the different fault types. The effectiveness of the proposed method was verified using the actual data from the wind turbine gearbox. In addition, a comparison with some existing methods demonstrates the superiority of this method for decoupling composite fault characteristics.

## 1. Introduction

Wind turbines, gas turbines and other advanced equipment are used widely in modern industry. The gearbox, a key component in these devices, is prone to failure when running under severe operating conditions such as heavy loads, large temperature differences, corrosive media, and alternating loads [1,2]. As the structure of machinery and equipment tends to be large and complex, the actual operation of the weak single fault will also have a chain reaction with the components of the transmission chain, resulting in various faults occurring in successive cascades to form a compound fault. Therefore, it is important to accurately and reliably extract fault features from vibration signals to achieve a composite fault diagnosis. In addition, this current problem is rarely covered in existing research, which mostly focuses on the compound failure caused by the local failure of the bearing and does not consider the compound failure caused by the bearing failure through the transmission path. The abovementioned situation is the starting point for this study and the basis for this paper.

Feature extraction aims at extracting feature information from the vibration signal to describe the operational status of the mechanical equipment. Over the past two decades, many scholars have explored the rotating machinery fault diagnosis field and introduced many diagnostic theories and methods. For example, empirical mode decomposition (EMD) [3,4], wavelet transform (WT) [5,6], variational mode decomposition fault (VMD) [7,8], and so on. Although these methods and their combination perform well on single faults, there are some limitations: for example, the existence of modal mixing in EMD [9,10], the diagnosis effectiveness of wavelet transform, which depends on the constant quality factor, and the choice of wavelet basis [11,12]. The lack of adaptiveness to complex signals and good separation and decoupling performance makes it impossible to effectively extract fault components from complex, random, and variable vibration signals.

The vibration signal collected by the sensor is regarded as a convolutional mixture of different excitation sources, fault sources and transmission channels, so the recovery of the fault signal can be considered a deconvolution process. In 2007, Endo et al. [13] successfully applied the minimum entropy deconvolution (MED) algorithm to the fault detection of rotating motors for the first time. MED can only highlight a small number of single pulses with high amplitude and cannot extract periodic pulses. In turn, the weak components in the composite fault cannot be extracted, while the filter after the MED iterative solution is not the optimal filter. Subsequently, McDonald et al. [14] developed a method named the maximum correlated kurtosis deconvolution (MCKD) algorithm, using the maximum value of correlated kurtosis as the iteration termination condition of the optimal filter. MCKD extracts periodic pulses while suppressing noise interference. He et al. [15] used MCKD to significantly enhance the periodic pulse component of the fault signal, which allowed the fault characteristics to be more prominent. Yang et al. [16] utilized MCKD as a pre-processing operation to highlight the continuous pulse component of the bearing. The noise interference is reduced while effectively improving the ability to represent the fault characteristics. However, the performance of the MCKD algorithm depends on three parameters: filter length, fault period, and shift number [17]. In addition, the fault period also needs to be rounded after resampling if it is not an integer. To overcome the shortcomings of MED and MCKD, McDonald and Zhao proposed Multipoint optimal minimum entropy deconvolution adjusted (MOMEDA) [18]. MOMEDA determines the period of fault occurrence by multipoint cliff values. At the same time, a non-iterative approach to obtaining the optimal filter enables the analysis of a non-integer number of fault cycles without resampling. Ma and Feng [19] redesigned the objective function of the MOMEDA algorithm based on the planetary bearing vibration signal characteristics and verified the effectiveness of the proposed method by numerical simulation and experimental analysis. Wang et al. [20] improved the ability of the MOMEDA algorithm to capture fault features by constructing an autoregressive mean shift model to improve noise immunity. Xiang et al. [21] combined MOMEDA and 1.5-dimensional Teager kurtosis spectrum analysis to effectively achieve feature extraction of composite bearing faults. Due to the coupling relationship between different fault characteristics, MOMEDA alone is not immune and thus leads to diagnostic failure when dealing with multiple faults.

In 2011, Selesnick proposed resonance-based sparse signal decomposition (RSSD) [22]. It is different from the traditional signal decomposition methods based on band division. The quality factor Q and redundancy degree r are flexibly selected to determine the basis function, which can effectively extract the periodic pulse characteristics in the fault signal without losing important information by waveform distortion. It solves the difficulty of separating fault features in traditional methods due to the similarity of decomposition frequencies. RSSD obtains a bank of base functions for high- and low-quality factors by the Tunable Q-Factor Wavelet Transform (TQWT) [23] according to the differences in oscillation properties of the signals (i.e., differences in the quality factor Q). Morphological Component Analysis (MCA) [24] is employed to decouple the signal into high and low resonance components with different quality factors. Thus, it realizes the separation effectively of the different elements in the unsteady signal. Traditional resonant sparse decomposition methods in which the quality factor Q and the redundancy factor r are defined by human selection [25,26] make the parameters more subjective and contingent. It will lead to the corresponding basis functions not being optimally matched to the transient shock components and harmonic elements, further reducing the decoupling effect. To address this issue, a parallel parameter optimized RSSD approach is proposed in this article. The compound indicator KHE serves as the objective function, such that the algorithm adaptively determines the values of the quality factor Q and the redundancy degree r based on the signal properties.

Motivated by the above discussion, a novel approach to wind turbine gearbox composite fault diagnosis based on MOMEDA and parallel parameter optimized RSSD is proposed in this article. Firstly, MOMEDA is used to deconvolute the signal to extract multiple periodic faults in the composite fault vibration signal. While achieving decoupling separation of the multi-fault, it effectively eliminates the influence of the transmission channels and external excitation sources. Secondly, the RSSD with parallel parameter optimized constructs the wavelet basis function bank to match the fault characteristics, which enables the interference components to be efficiently suppressed in the decoupled fault signal and the weak fault pulse to be enhanced. The superiority and effectiveness of the proposed approach are verified using the measured signals of the wind turbine gearbox.

The main structure of this article is composed as follows: Section 2 describes the theories of MOMEDA. Section 3 introduces the parallel parameter optimized RSSD based on WOA. Section 4 presents the detailed steps for extracting multi-fault characteristics with the proposed method. In Section 5, the actual composite fault signals are collected from wind turbine gearboxes to verify the proposed method and introduce a quantitative index to evaluate the performance between our approach and two comparison approaches to prove the superiority of the method in this article. Finally, the conclusions of this study are summarized in Section 6.

## 2. Multipoint Optimal Minimum Entropy Deconvolution Adjusted

MOMEDA utilizes a target vector to define the position and weight of the pulse sequence to be solved and applies multi-point kurtosis values to determine the period of fault occurrence. Multiple pulse target identification and deconvolution algorithms are implemented at determined locations to obtain continuous periodic pulse components.

When a rotating machine fails, the impulse signal x is modulated to s by the system transmission path response h, and together with the noise q is collected by the sensor to form the vibration signal y. The process is expressed as:

(1) y=s+q=h∗x+q

The impulse signal *x* is recovered from the vibration signal *y* by an optimal filter. The MOMEDA solving process for the optimal filter can transform into a search for the maximum value of the multipoint D-parameter, using the multipoint D-parameter to reflect the shock characteristics of the filtered signal, and the related expressions are defined as follows:

(2) $$\mathrm{MDN}(\vec{y},\vec{t})=\frac{1}{\left\|\vec{t}\right\|}\frac{{\vec{t}}^{T}\vec{y}}{\left\|\vec{y}\right\|}$$

(3) $$\mathrm{MOMEDA}:\underset{\vec{f}}{\max}MDN(\vec{y},\vec{t})=\underset{\vec{f}}{\max}\frac{{\vec{t}}^{T}\vec{y}}{\left\|\vec{y}\right\|}$$

where the target vector $\vec{t}$ is a constant that defines the position and weight of the pulse sequence to be deconvoluted; $\vec{f}$ denotes the filter coefficients.

The extreme value of Equation (3) is obtained by taking the derivative of the filter coefficient ($\vec{f}=f_{1},f_{2},\ldots ,f_{L}$).

(4) $$\frac{d}{d\vec{f}}(\frac{{\vec{t}}^{T}\vec{y}}{\left\|\vec{y}\right\|})=\frac{d}{d\vec{f}}\frac{t_{1}y_{1}}{\left\|\vec{y}\right\|}+\frac{d}{d\vec{f}}\frac{t_{2}y_{2}}{\left\|\vec{y}\right\|}+\ldots +\frac{d}{d\vec{f}}\frac{t_{N-L}y_{N-L}}{\left\|\vec{y}\right\|}$$

where, $\frac{d}{d\vec{f}}\frac{t_{k}y_{k}}{\left\|\vec{y}\right\|}=\|\vec{y}{\|}^{-1}t_{k}{\vec{M}}_{k}-\|\vec{y}{\|}^{-3}t_{k}y_{k}X_{0}\vec{y}$, ${\vec{M}}_{k}=\left[\begin{array}{c} x_{k+L-1}\\ x_{k+L-2}\\ \vdots \\ x_{k} \end{array}\right]$. Hence, Equation (4) can be written in the following form:

(5) $$\frac{d}{d\vec{f}}(\frac{{\vec{t}}^{T}\vec{y}}{\left\|\vec{y}\right\|})=\|\vec{y}{\|}^{-1}(t_{1}{\vec{M}}_{1}+t_{2}{\vec{M}}_{2}+\ldots +t_{N-L}{\vec{M}}_{N-L})-\|\vec{y}{\|}^{-3}{\vec{t}}^{T}\vec{y}X_{0}\vec{y}$$

It can be further simplified as follows:

(6) $$t_{1}{\vec{M}}_{1}+t_{2}{\vec{M}}_{2}+\ldots +t_{N-L}{\vec{M}}_{N-L}=X_{0}\vec{t}$$

Solving the extreme value with Equation (5) to zero, we can obtain:

(7) $$\begin{array}{c} \|\vec{y}{\|}^{-1}X_{0}\vec{t}-\|\vec{y}{\|}^{-3}{\vec{t}}^{T}\vec{y}X_{0}\vec{y}=0\\ \frac{{\vec{t}}^{T}\vec{y}}{\|\vec{y}{\|}^{2}}X_{0}\vec{y}=X_{0}\vec{t} \end{array}$$

Owing to $\vec{y}={X_{0}}^{T}\vec{t}$ and the assumed existence of ${\left(X_{0}{X_{0}}^{T}\right)}^{-1}$, that:

(8) $$\frac{{\vec{t}}^{T}\vec{y}}{\|\vec{y}{\|}^{2}}\vec{f}={\left(X_{0}{X_{0}}^{T}\right)}^{-1}X_{0}\vec{t}$$

Since the multiples of $\vec{f}$ are also the solutions of Equation (9). So, the multiples of $\vec{f}={\left(X_{0}{X_{0}}^{T}\right)}^{-1}X_{0}\vec{t}$ are also the solutions of MOMEDA. The advantage of this solution is to avoid iterative operations, Thus, the MOMEDA filter and output solution can be summarized as follows:

(9) $$\vec{f}={\left(X_{0}{X_{0}}^{T}\right)}^{-1}X_{0}\vec{t}$$

(10) $$X_{0}={\left[\begin{array}{cccccc} x_{L} & x_{L+1} & x_{L+2} & \cdots & \cdots & x_{N}\\ x_{L-1} & x_{L} & x_{L+1} & \cdots & \cdots & x_{N-1}\\ x_{L-2} & x_{L-1} & x_{L} & \cdots & \cdots & x_{N-2}\\ \vdots & \vdots & \vdots & \ddots & \cdots & \vdots \\ x_{1} & x_{2} & x_{3} & \cdots & \cdots & x_{N-L+1} \end{array}\right]}_{L\;\mathrm{by}\;N-L+1}$$

(11) $$\vec{y}={X_{0}}^{T}\vec{f}$$

When performing target location and fault detection with MOMEDA, a chain of impulses with fault period T as the step is added to the target vector.

(12) $$\begin{array}{c} t_{n}=P_{n}(T)=\delta_{round(T)}+\delta_{round(2T)}+\delta_{round(3T)}+\ldots ,\\ \vec{t}=\vec{P}(T) \end{array}$$

where, δ denotes the pulse of sample n. The non-integer period T should be rounded to the integer value closest to the fault pulse.

MOMEDA introduces Multipoint Kurtosis (MK) as a measure of fault characteristics based on kurtosis. When the output result $\vec{y}$ matches the multiple of $\vec{t}$, we obtain the standardized MK equation as follows:

(13) $$\mathrm{Multipoint}\;\mathrm{Kurtosis}=\frac{{\left(\sum_{n=1}^{N-L}t_{n}^{2}\right)}^{2}}{\sum_{n=1}^{N-L}t_{n}^{8}}\frac{\sum_{n=1}^{N-L}{\left(t_{n}y_{n}\right)}^{4}}{\sum_{n=1}^{N-L}{\left(y_{n}^{2}\right)}^{2}}$$

## 3. The Parallel Parameter Optimized RSSD Base on WOA

### 3.1. Tunable Q-Factor Wavelet Transform

**Figure 1:**
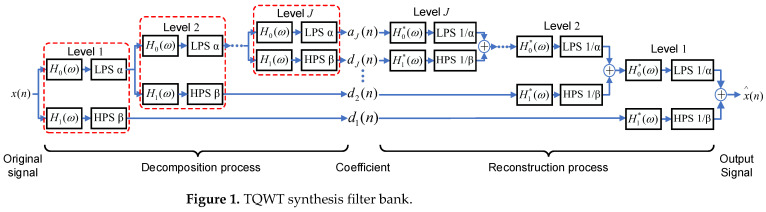
TQWT synthesis filter bank.

TQWT breaks through the disadvantage of the constant quality factor of the traditional wavelet transform and makes the selection of basis function more flexible by selecting quality factor Q and redundancy factor r, which can better match the signals with different vibration properties. TQWT uses a dual-channel filter bank to decompose the signal into multiple scales and obtain the transform coefficients by layer-by-layer decomposition. Thus, it obtains a sparse representation of the high and low resonance components. The TQWT filter bank is clarified in Figure 1. LPS and HPS denote the low-pass scale transform and high-pass scale transform, respectively. α and β are the low-pass scale factor and high-pass scale factor.

To achieve perfect reconstruction, the frequency response function $H_{0}(\omega )$ of the low-pass filter and the frequency response function $H_{1}(\omega )$ of the high-pass filter are defined as follows:

(14) $$H_{0}(\omega )=\left\{ \begin{array}{ll} 1 & \left|\omega \right|\leq (1-\beta )\pi \\ \theta \left(\frac{\omega +(\beta -1)\pi }{\alpha +\beta -1}\right) & \;\;(1-\beta )\pi <\left|\omega \right|<\alpha \pi \\ 0 & \alpha \pi \leq \left|\omega \right|\leq \pi \end{array}\right.$$

(15) $$H_{1}(\omega )=\left\{ \begin{array}{cc} 1 & \left|\omega \right|\leq (1-\beta )\pi \\ \theta \left(\frac{\alpha \pi -\omega }{\alpha +\beta -1}\right) & (1-\beta )\pi <\left|\omega \right|<\alpha \pi \\ 0 & \alpha \pi \leq \left|\omega \right|\leq \pi \end{array}\right.$$

where, $\theta (\omega )=0.5(1+\cos\omega )\sqrt{2-\cos\omega }$, |ω|≤π 0<α<1, 0<β≤1, α+β>1. Equation (16) shows the relationship between the scale transformation factor (α,β) and the TQWT parameters (Q,r), and Equation (17) provides the required number of decomposition layers.

(16) $$\beta =\frac{2}{Q+1},\alpha =1-\frac{\beta }{r}$$

(17) $$J_{\max}=\left\lfloor \frac{\log(N/4(Q+1))}{\log((Q+1)/(Q+1-2)/r)}\right\rfloor$$

where: N is the signal length and ⎣•⎦ is the downward rounding sign.

The high resonance components $x_{1}$ and low resonance components $x_{2}$ are extracted from the original vibration signal, and the relevant expressions are as follows:

(18) $$y=x_{1}+x_{2}$$

Achieving sparse representation of different components of the signal by MCA. The objective function of extracting the different components is expressed as follows:

(19) $$\begin{array}{l} \underset{\omega_{1},\omega_{2}}{\arg\min}\;\sum_{j=1}^{j_{1}+1}\lambda_{1,j}{\left\|\omega_{1,j}\right\|}_{1}+\sum_{j=1}^{j_{2}+1}\lambda_{2,j}{\left\|\omega_{2,j}\right\|}_{1}\\ x={\mathrm{TQWT}}_{1}^{-1}(\omega_{1})+{\mathrm{TQWT}}_{2}^{-1}(\omega_{2}) \end{array}$$

where $\omega_{i,j}$ is the sub-band j of TQWT(i=1,2). $\lambda_{1}$, $\lambda_{2}$ is the regular term parameters.

However, the vibration signal inevitably has interference by background noise in actual works, and the signal separation will be transformed from Equation (19) as follow:

(20) $$y=x_{1}+x_{2}+x_{noise}$$

(21) $$\underset{\omega_{1},\omega_{2}}{\arg\min}{\left\|y-\Phi_{1}\omega_{1}-\Phi_{2}\omega_{2}\right\|}_{2}^{2}+\sum_{j=1}^{j_{1}+1}\lambda_{1,j}{\left\|\omega_{1,j}\right\|}_{1}+\sum_{j=1}^{j_{2}+1}\lambda_{2,j}{\left\|\omega_{2,j}\right\|}_{1}$$

where $\Phi_{1}$ and $\Phi_{2}$ denote the inverse wavelet transform of high Q-factors and low Q-factors, respectively. Then, the minimum value of the above Equation (21) is solved to obtain the high and low resonance components according to Equation (22).

(22) $$x_{n}={\mathrm{TQWT}}^{-1}(\omega_{n}),\;n=1,2$$

### 3.2. Whale Optimization Algorithm

Whale Optimization Algorithm (WOA) is a novel intelligent optimization algorithm proposed by Mirjalili in 2016 [27]. The optimal solution is solved via imitating the predatory behavior of whales. The algorithm consists of three processes.

(1) Surrounding the prey

Assuming that the population size is NP, and the dimension is D, the position of the ith whale in the D-dimensional space is $X_{i}=(x_{i}^{1},x_{i}^{2},\ldots x_{i}^{D})$, i=1,2,…NP. The algorithm supposes that the desired problem is the optimal position of the whale, and the other whales will be updated and adjusted oriented to this optimal position. The mathematical model is expressed as follows:

(23) $$X(t+1)=X^{*}(t)-A\cdot D$$

(24) $$D=\left|C\cdot X^{*}(t)-X(t)\right|$$

(25) $$A=2a\cdot r_{1}-a$$

(26) C=2·r

(27) $$a=2(1-t/T_{\max})$$

where D denotes the distance between the individual position and the optimal position; A is the convergence factor. $X^{*}(t)$ is the optimal position at t iterations, X(t) is the individual position at t iterations, $r_{1}$ and $r_{2}$ are random numbers between [0, 1], $T_{\max}$ is the maximum number of iterations, and a decreases linearly from 2 to 0 with the number of iterations.

(2) Bubbling net attack

Shrinkage encircling: The convergence factor A decreases linearly with a to achieve position update. When the value of A is taken between [−1, 1], the position of each individual is between X(t) and $X^{*}(t)$ as a means to achieve prey bracketing.

Spiral updating position: whales spit out bubbles of different sizes for feeding while swimming towards the best position in a spiral posture. The mathematical model is

(28) $$X(t+1)=D^{\prime }\cdot e^{bl}\cdot \cos(2\pi l)+X^{*}(t)$$

where: $D^{\prime }$ denotes the distance between the individual of the t iterations and the current optimal solution. l is a random number between [−1, 1], b is the spiral shape constant.

During the process of prey encirclement, the whales shrink to surround and spiral forward simultaneously, and the probability of occurrence is 50% for either mode of travel.

(29) $$X(t+1)=\{\begin{array}{ll} X^{*}(t)-A\cdot D & \;\mathrm{if}\;p<0.5\\ D^{\prime }\cdot e^{bl}\cdot \cos(2\pi l)+X^{*}(t) & \;\mathrm{if}\;p\geq 0.5 \end{array}$$

(3) Searching for prey

The whales will stop approaching the best whale individual in this stage and instead update their position by randomly searching a large area to approach any whale individual. In this case, the value of A is taken as |A|>1. This predation strategy will cause the current individual to deviate from the target prey but will enhance the global search ability and avoid falling into local optimum. The mathematical model as follow:

(30) $$D=\left|C\cdot X_{rand}(t)-X(t)\right|$$

(31) $$X(t+1)=X_{rand}(t)-A\cdot D$$

where $X_{rand}$ denotes the location of random individuals in the current population.

### 3.3. Design Objective Function

Kurtosis is a 4th order statistic that reflects the sharpness of the waveform for the random variable and is sensitive to the impulse component of the signal, which is defined as:

(32) $$K=\frac{E(x-\mu )}{\sigma^{4}}$$

Information entropy represents the uncertainty of the source information and the randomness of the event occurrence, and its value is only related to the probability distribution of the variables. Suppose a source $X=\{x_{1},x_{2},\ldots ,x_{N}\}$ consists of a discrete random variable that the probability of occurrence is $p_{i}=P(x_{i})(i=1,2,\cdots N)$ and $\sum_{i=1}^{N}p=1$, then the information entropy of the source X is expressed as:

(33) $$H(X)=-\sum_{i=1}^{N}p_{i}\ln p_{i}$$

where $\underset{p\to 0}{\lim}p\ln(p)=0$, the more balance the distribution of variables in the source, the greater the value of information entropy. The envelope spectrum is combined with the information entropy, i.e., the envelope spectrum entropy [28]. The fault signal is treated as a signal source after envelope spectrum processing, and the frequency amplitude of each point is regarded as a variable in the signal source. The formula for calculating the envelope spectral entropy is as follows:

(34) $$H_{e}=-\sum_{i=1}^{N}p_{k}\ln p_{k}$$

where $p_{k}$ represents the envelope spectrum of the vibration signal. The envelope spectrum entropy can measure the uniformity of frequency distribution in the envelope signal and can express the complexity of the signal in the envelope domain.

In this article, we combine kurtosis and envelope spectral entropy, kurtosis as an indicator of time-domain feature can describe the impulsiveness of the signal, and envelope spectral entropy as an indicator of frequency-domain feature can represent the strength of periodic pulses. A composite indicator is constructed to reflect the time-frequency domain properties and the expression is as follows:

(35) $$KHE=\frac{K}{H_{e}}$$

This indicator possesses the advantages of kurtosis and envelope spectral entropy, and it can measure the impulsivity and periodicity of the signal at the same time. The more prominent the impulsivity and periodicity of the signal, the larger the value of the indicator.

Overall, the parallel parameter optimized RSSD based on WOA as proposed in this article implements the process as follows:

(1) The parameters of the algorithm are determined: population size NP, population dimension Dm, and the maximum number of iterations $T_{\max}$. For resonant sparse decomposition, it is required to find the optimal four parameters: $Q_{H}$, $Q_{L}$, $r_{H}$, $r_{L}$, so the dimension is set to Dm = 4, the population size set to NP = 30, $T_{\max}$ = 50.
(2) Population initialization: The optimal parameters should be bounded, and the correlation between quality factors should be as low as possible. The value range of $Q_{1}$ takes as [8,15], the value range of $Q_{2}$ takes as [1,3], and the value range of redundancy factors $r_{1}$ and $r_{2}$ take as [2,5]. Then, to reduce the calculation, the accuracy of the four parameters is reserved to one single decimal.
(3) The objective function value is calculated: the composite index constructed by kurtosis and envelope spectral entropy serves as the objective function. The objective function value of an individual is calculated and the current optimal individual is determined.
(4) The main loop of the algorithm is entered: if p<0.5 and |A|<1, the individual updates the current position by Equation (23), otherwise the individual position is updated by Equation (31). When p≥0.5, the position is updated according to Equation (29).
(5) Evaluating the whole whale population and iterative optimization until the algorithm converges, it obtains the optimal objective function value KHE. Obtain the RSSD parameters after parallel optimization: $Q_{H}$, $Q_{L}$, $r_{H}$, $r_{L}$.

## 4. The Procedure of Compound Fault Diagnosis

**Figure 2:**
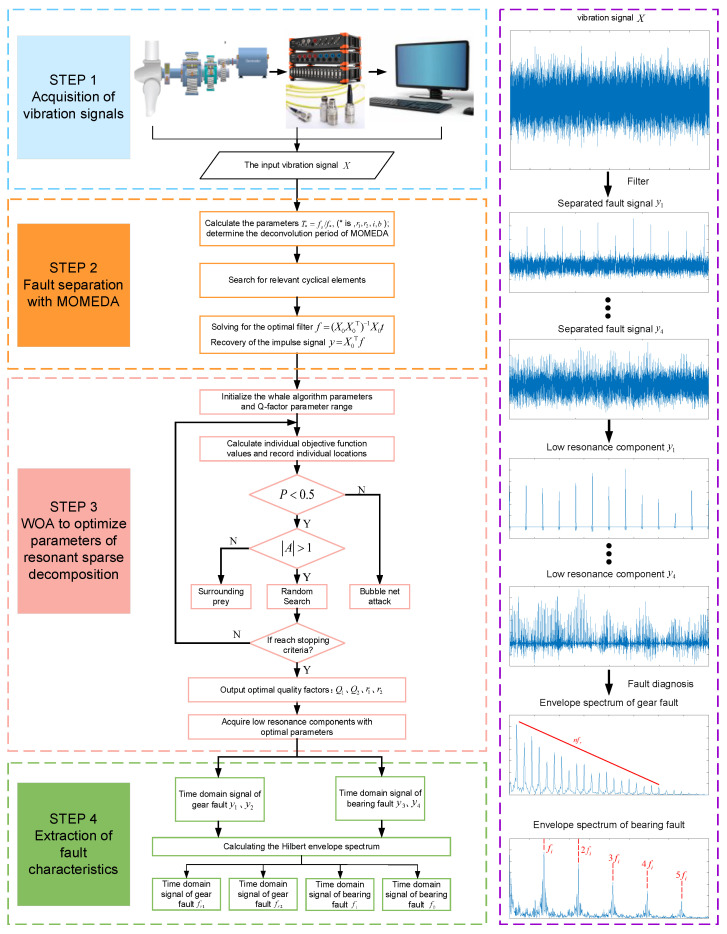
The flow chart of the proposed paper.

The method proposed in this article is suitable to separate and extract the compound faults of the gear faults and the bearing faults from the wind turbine gearbox. Firstly, the input vibration signal is pre-processed, the deconvolution period is set according to the fault frequency of the damaged part, and the vibration signal is decoupled into a single fault by MOMEDA. Secondly, the low resonance component is decomposed from the pre-processed signal with optimized RSSD. Finally, the envelope analysis of the low-resonance components is applied to extract the fault characteristic frequency. The flowchart of the method is shown in Figure 2.

## 5. Application of Proposed Method

### 5.1. Experiment Introduction

**Figure 3:**
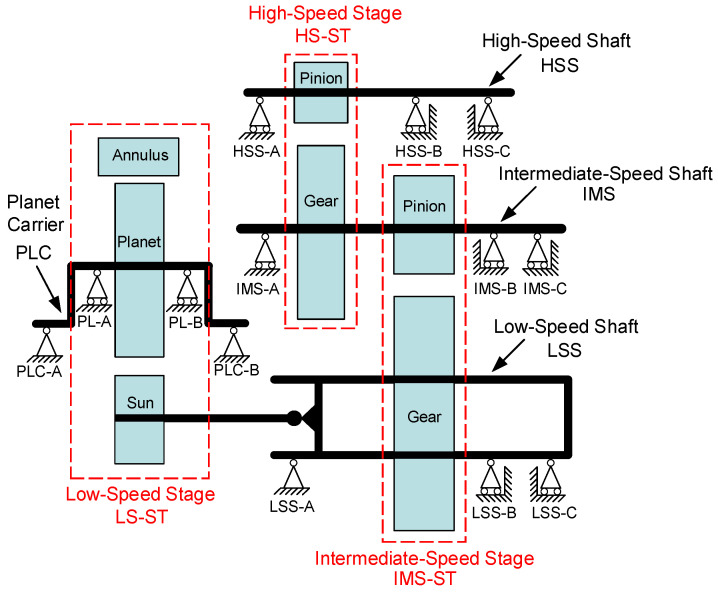
Gearbox internal layout and nomenclature abbreviations.

In this paper, 750 kW wind turbine gearbox data provided by The National Renewable Energy Laboratory (NREL) [29] were used to verify the performance of the proposed method. The gearbox first finished run-in in the NREL dynamometer facility and then was sent to the wind plant for testing under actual operating conditions. The gearbox high-speed shaft is operated at 1800 rpm during the test, and the sensors collect vibration acceleration signals with a sampling frequency of 40 kHz. The gearbox consists of a low-speed stage planetary gearbox and a parallel shaft gearbox and has an overall transmission ratio of 1:81.491. Figure 3 gives the internal layout and nomenclature abbreviations of the gearbox.

**Figure 4:**
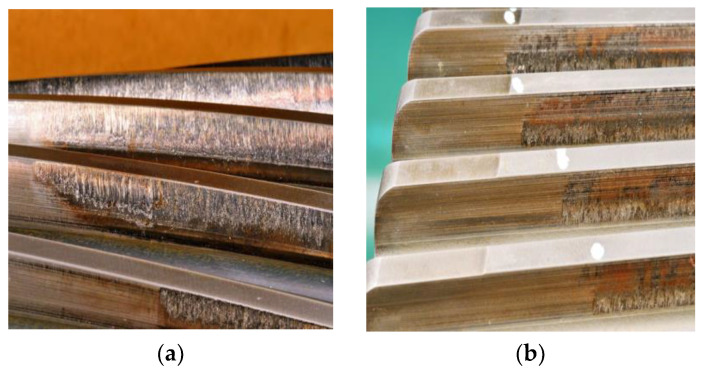

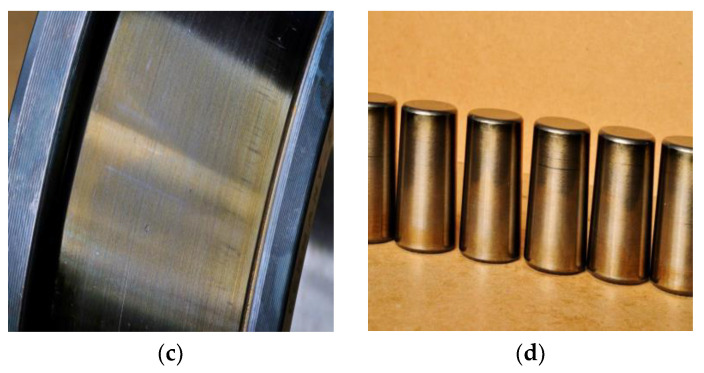
Gear and bearing failure pictures. (**a**) High-speed shaft pinion fault; (**b**) Intermediate speed shaft gear fault; (**c**) Bearing inner ring fault; (**d**) Bearing rollers fault.

The gearbox experienced two oil loss events during the actual test, where it damaged its internal bearings and gear components. The damaged gearbox was disassembled for fault analysis, and it was found that the large and small gears of the high-speed gear pair in the gearbox were seriously scuffed, and the inner ring races of the bearings and the two ends of the rolling bodies were overheated. The failure of each component is shown in Figure 4.

The configuration parameters of the gearbox are listed in Table 1. The experiment takes the HSS pinion failure, the IMS large gear, and the HSS bearing C as the research targets. The failed bearing for this experiment is SKF 3222 J2, and Table 2 describes its structural parameters. Table 3 provides the results of the failure frequency calculations for the damaged parts.

### 5.2. Experiment Analysis

**Figure 5:**
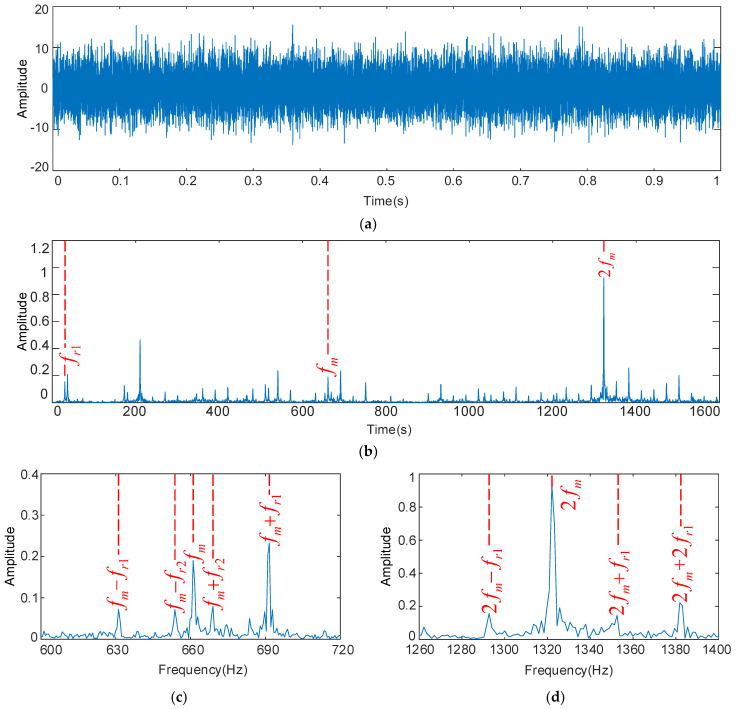

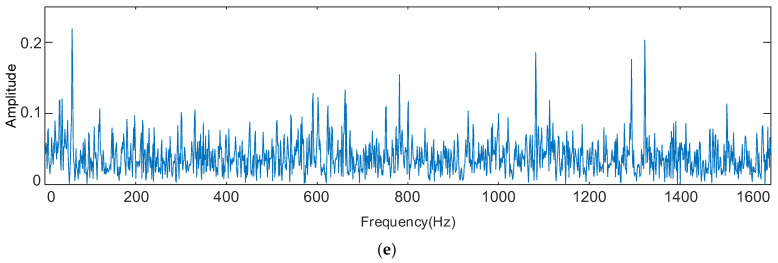
Composite fault signal of wind turbine gearbox and its frequency spectrum. (**a**) Time domain; (**b**) Fourier spectrum (**c**) 600–720 Hz spectrum amplification; (**d**) 1240–1400 Hz spectrum amplification; (**e**) Envelope spectrum.

Figure 5 shows the results of the analysis of the raw vibration signal of the gearbox compound fault. In Figure 5a, the pulse component triggered by faults has been completely submerged in the noise and failed to detect the periodical components. As seen from Figure 5b, the frequency components are mostly concentrated on the meshing frequency $f_{m}$ and its multiplier $2f_{m}$ around, and the rotational frequency component $f_{r1}$ is relatively weak. In the amplified plots of the original signal spectrum in Figure 5c,d, the fault characteristic information can see: centered on the meshing frequencies $f_{m}$ and $2f_{m}$, with $f_{m}\pm f_{r1}$, $2f_{m}\pm f_{r1}$, and $2f_{m}+2f_{r1}$ as the sidebands. Hence, it can be concluded that the HSS pinion is faulty. In Figure 5c, the fault feature component centered on the meshing frequency $f_{m}$ and with $f_{m}\pm f_{r2}$ as the sidebands exist, but the spectrum is non-obvious. Meanwhile, there is no fond of the feature components associated with the IMS gear in Figure 5d. Therefore, it is considered a possibility that the fault is in the IMS gear. The feature components reflect the gear failures and bearing failures uncovered in the envelope spectrum shown in Figure 5e.

**Figure 6:**
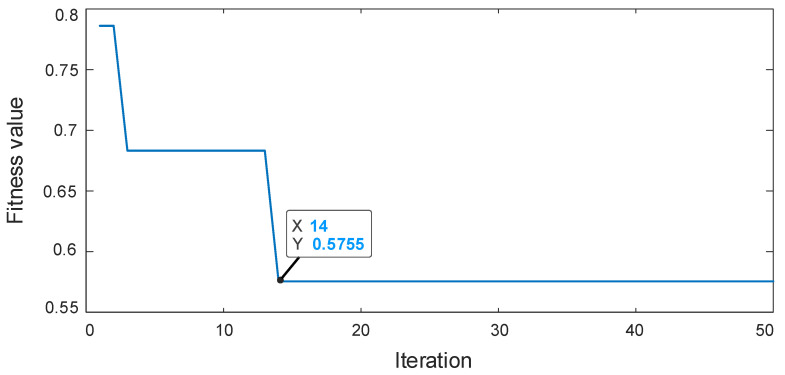
The RSSD optimized convergence curve.

**Figure 7:**
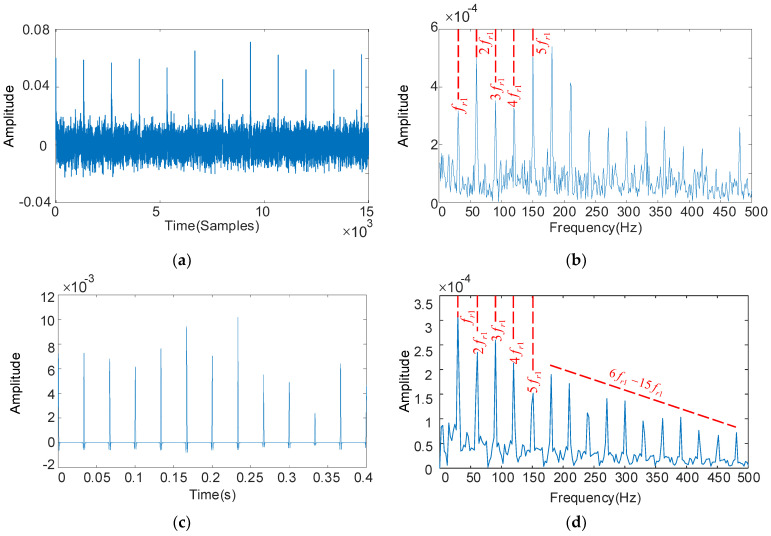
The decoupled pinion vibration signal and its frequency spectrum: deconvolution results of MOMEDA: filtered time domain waveforms and their frequency spectrum (**a**,**b**); Results obtained by MOMEDA and optimized RSSD: time domain waveforms and their frequency spectrum (**c**,**d**).

The deconvolution period $T_{r1}$ is set with the fault characteristics of the HSS pinion. The RSSD optimized convergence curve is shown in Figure 6. When the number of iterations reaches 14, the algorithm converges to 0.5755. The parameters obtained by optimization are: $Q_{H}$ = 4.1, $Q_{L}$ = 1.0, $r_{H}$ = 5.0, $r_{L}$ = 3.5. The rest of the optimization process is similar and will not be listed later. The periodic pulses are visible in Figure 7a, and the remarkable fault frequency characteristics with $f_{r1}$ as the interval can be seen in Figure 7b, but $f_{r1}$, $3f_{r1}$, and $4f_{r1}$ fault frequencies are relatively low. The low resonance components obtained by substituting the optimized parameters into RSSD are shown in Figure 7c. we can see that the periodic pulse characteristics are enhanced and the interference is suppressed effectively. From Figure 7d, we can see that the $f_{r1}$–$5f_{r1}$ and $6f_{r1}$–$15f_{r1}$ failure frequencies provide evidence of degradation and decay of the high-speed shaft pinion, thus diagnosing the presence of the HSS pinion faulty.

**Figure 8:**
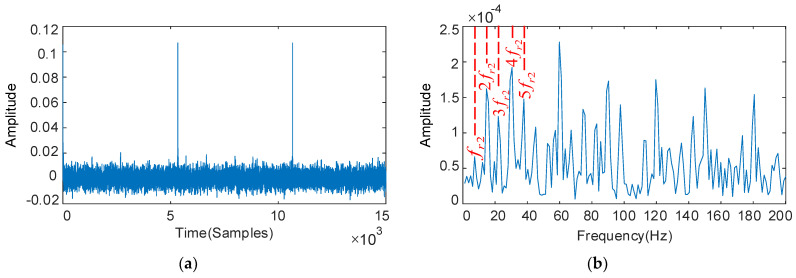

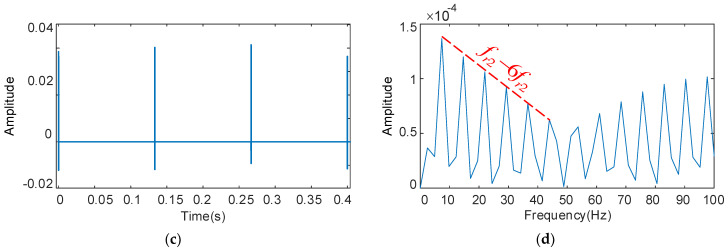
The decoupled gear vibration signal and its frequency spectrum: deconvolution results of MOMEDA: filtered time domain waveforms and their frequency spectrum (**a**,**b**); Results obtained by MOMEDA and optimized RSSD: time domain waveforms and their frequency spectrum (**c**,**d**).

The deconvolution period $T_{r2}$ is set with the fault characteristics of the IMS gear, and the results as shown in Figure 8. Periodic pulses can be seen in Figure 8a, and the rest of the fault characteristics are not apparent except for $4f_{r2}$ in Figure 8b, but $4f_{r2}$ is easily confused with the HSS pinion fault characteristic frequency $f_{r1}$, leading to misdiagnosis. The optimized parameters are: $Q_{H}$ = 4.0, $Q_{L}$ = 1.2, $r_{H}$ = 5.0, $r_{L}$ = 3.5, and the low-resonance components obtained by decomposition are shown in Figure 8c,d. The interference components are reduced in the time domain diagram, and the IMS gear fault characteristic frequency $f_{r2}$ and its multiples $2f_{r2}$–$6f_{r2}$ predominate in the envelope spectrum. The resonance decomposition suppresses the interference obviously, and further extracts and enhances the weak fault features.

**Figure 9:**
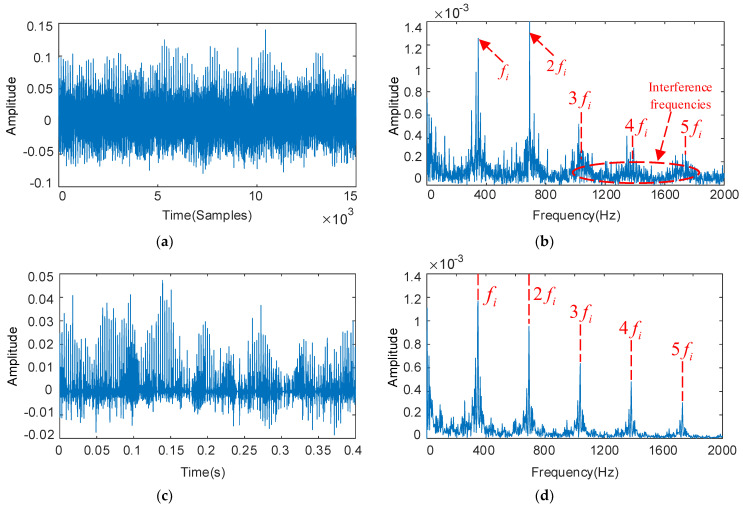
The decoupled bearing inner ring vibration signal and its frequency spectrum: deconvolution results of MOMEDA: filtered time domain waveforms and their frequency spectrum (**a**,**b**); Results obtained by MOMEDA and optimized RSSD: time domain waveforms and their frequency spectrum (**c**,**d**).

The deconvolution period $T_{i}$ is set with the fault characteristic frequency of the bearing inner ring, and the results as shown in Figure 9. From Figure 9b, we can see serious interference around the fault signature frequencies $3f_{i}$, $4f_{i}$, and $5f_{i}$, which is unable to identify the fault signature. The optimized parameters are $Q_{H}$ = 14.2, $Q_{L}$ = 2.7, $r_{H}$ = 3.5, $r_{L}$ = 3.5, and the low-resonance components obtained by decomposition as shown in Figure 9c,d. The periodicity of the fault signal in the time domain is enhanced, the feature frequency $f_{i}$ of the bearing inner ring fault and its multiplication frequency $2f_{i}$–$5f_{i}$ in the envelope spectrum became more significant, and the interference components become well suppressed.

**Figure 10:**
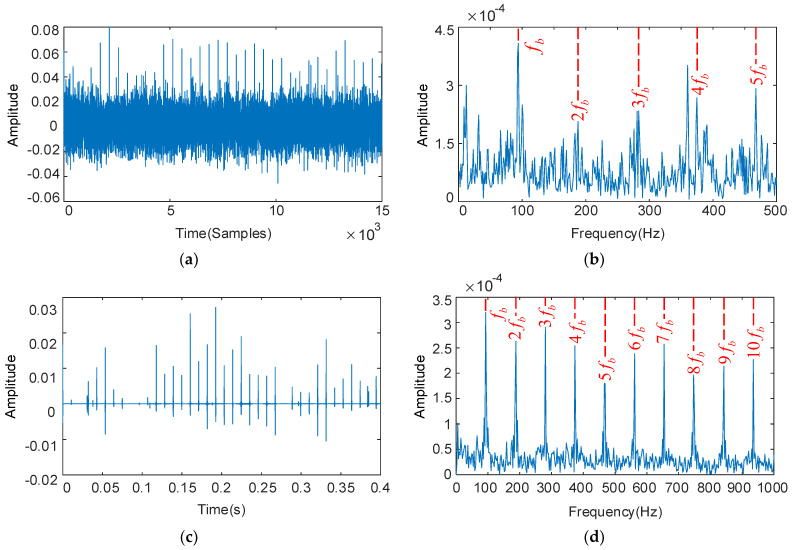
The decoupled bearing rollers vibration signal and its frequency spectrum: deconvolution results of MOMEDA: filtered time domain waveforms and their frequency spectrum (**a**,**b**); results obtained by MOMEDA and optimized RSSD: time domain waveforms and their frequency spectrum (**c**,**d**).

The deconvolution period $T_{b}$ is set with the fault characteristic frequency of the bearing rollers, and the results as shown in Figure 10. The envelope spectrum in Figure 10b shows that the fault feature frequencies $2f_{b}$, $3f_{b}$, and $4f_{b}$ are not apparent and the bearing fault characteristics cannot be identified. The optimized parameters are $Q_{H}$ = 8.0, $Q_{L}$ = 2.0, $r_{H}$ = 3.5, $r_{L}$ = 3.5, and the low-resonance components obtained by decomposition as shown in Figure 10c,d. Though some impulse components are missing in the time domain diagram, the overall periodic characteristics are remarkably improved, and the interference components effectively removed. The high-speed grade bearing rollers fault characteristic frequency $f_{b}$ and its multiples $2f_{b}$–$10f_{b}$ dominate significantly in the envelope spectrum, and the interference component restraining effect is obvious.

In summary, the analysis results show that the proposed method can not only successfully decouple and separate various types of fault characteristics from the composite fault signal, but also has a remarkable suppression effect for interferences, further highlighting the periodicity and impulsiveness of fault characteristics, which helps to improve the accuracy and reliability of wind turbine gearbox composite fault diagnosis.

### 5.3. Comparative Analysis

The superiority and effectiveness of the proposed method are verified through a comparative analysis using the two methods. The MCKD algorithm in Reference [14] and the improved MCKD algorithm in Reference [16] are used to analyze the composite fault signals.

**Figure 11:**
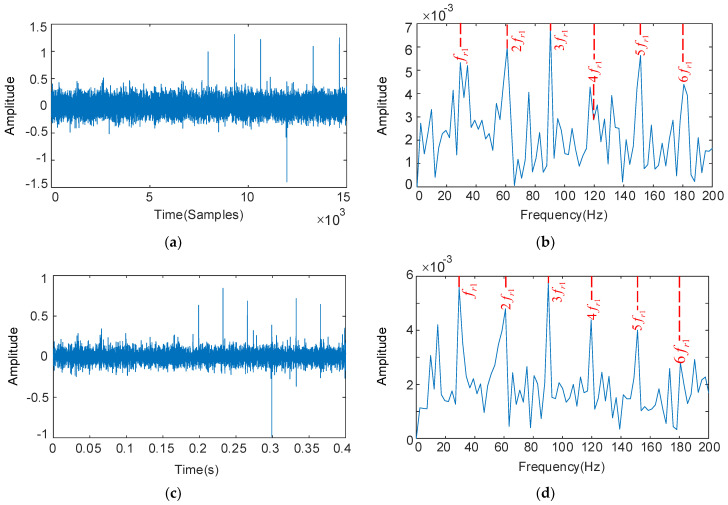
The HSS pinion deconvolution results by MCKD: the filtered time domain waveform and their spectrum (**a**,**b**); results obtained by MCKD + RSSD: time domain waveforms and their frequency spectrum (**c**,**d**).

**Figure 12:**
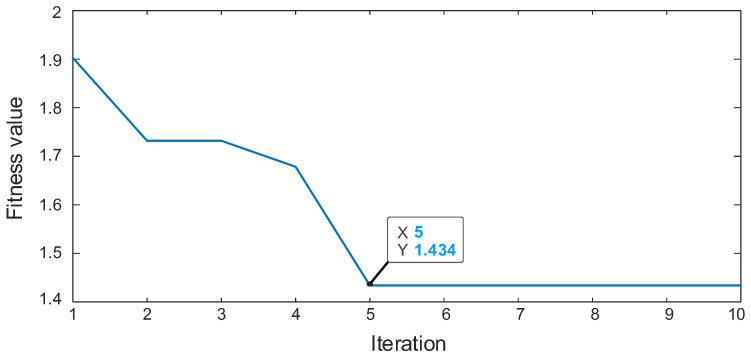
The RSSD optimized convergence curve of comparative method.

The two methods mentioned above are used to decouple the HSS pinion fault from the composite fault signal and analyze and process it. The parameters are set as follows: number M = 5 is shifted, length L = 500 is filtered, the above calculation results of the fault frequency are introduced, and the analysis results are shown in Figure 11. From the time domain plot in Figure 11a, the MCKD algorithm can extract only a limited set of pulses because the result after deconvolution is not an iterative optimal solution. The resonant sparse decomposition was used for further processing, the RSSD optimized convergence curve is shown in Figure 12. When the number of iterations reach 5, the algorithm converges to 1.434. The parameters obtained by optimization are: $Q_{H}$ = 8.3, $Q_{L}$ = 3, $r_{H}$ = 5, and $r_{L}$ = 3.4. From the time domain plot in Figure 11c, though it removes part of the noise interference, the continuous periodic pulse sequence cannot be extracted subject to the algorithm performance of MCKD.

The envelope spectrum analysis shown in Figure 11b, the fault characteristic frequency $f_{r1}$ and its multiples $4f_{r1}$ do not prominent in the envelope spectrum. The fault feature frequencies dominate in the frequency spectrum with the optimized resonance decomposition from Figure 11d. The fault characteristics of the HSS pinion are accurately identified.

**Figure 13:**
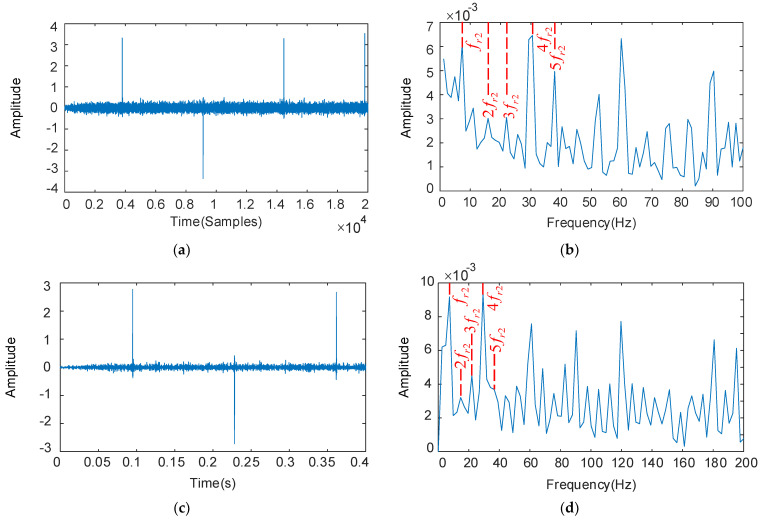
The IMS gear deconvolution results by MCKD: the filtered time domain waveform and their spectrum (**a**,**b**); the results obtained by MCKD + RSSD: the filtered time domain waveform and their spectrum (**c**,**d**).

The same procedure is applied for the IMS gear, and the parameters obtained with the optimized RSSD are: $Q_{H}$ = 8.2, $Q_{L}$ = 2.9, $r_{H}$ = 5, and $r_{L}$ = 3.5. Some of the disturbances are removed in the time domain as shown in Figure 13. However, it failed to extract the fault feature frequency of the IMS gear effectively due to the performance of MCKD.

**Figure 14:**
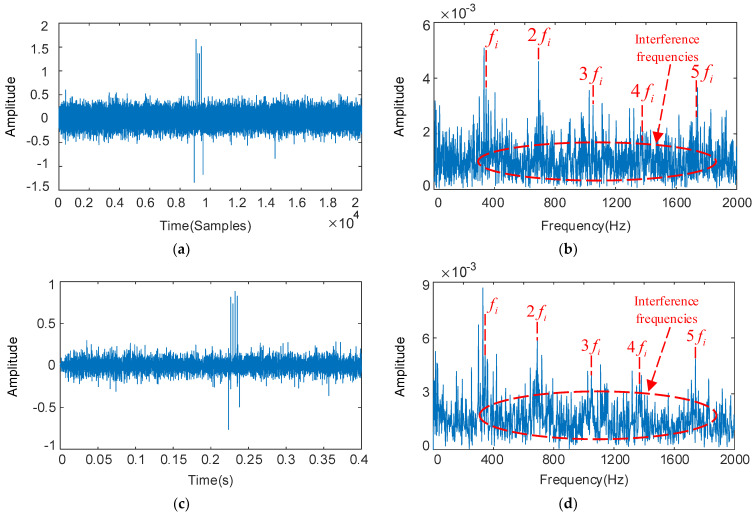
The bearing inner ring deconvolution results by MCKD: the filtered time domain waveform and their spectrum (**a**,**b**); results obtained by MCKD + RSSD: time domain waveforms and their frequency spectrum (**c**,**d**).

**Figure 15:**
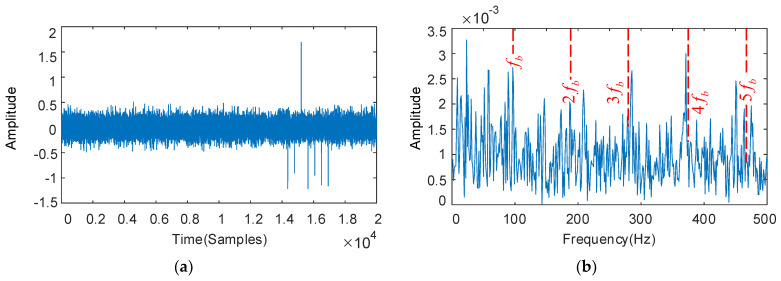

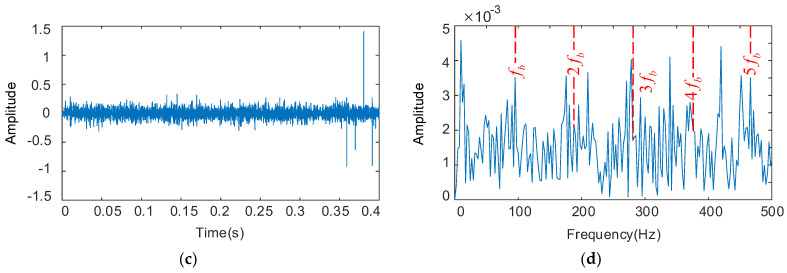
The bearing rollers deconvolution results by MCKD: the filtered time domain waveform and their spectrum (**a**,**b**); results obtained by MCKD + RSSD: time domain waveforms and their frequency spectrum (**c**,**d**).

The bearing inner ring fault signals and bearing rollers fault signals are directly processed with the optimized parameters of the method proposed in this paper, so as to imitate the process of artificially selecting parameters in traditional resonance decomposition. The results are shown in Figure 14 and Figure 15. It can be seen from the spectrum that the traditional resonance decomposition has subjectivity and randomness due to the artificial selection of parameters, which means that the basis function cannot effectively match the fault pulse components. The above comparative analysis further illustrates the validity and superiority of the proposed method in this paper.

To evaluate the performance of extracting fault features quantitatively, the fault feature coefficient (FFC) [30] is introduced as a quantitative index to select the fault components. The larger the FFC value, the more adequate the periodic pulse information contained in the fault frequency component. The FFC is defined as follows:

(36) $$FFC=\frac{\sum {\left[A(f)\right]}^{2}}{\sum {\left[A(f)\right]}^{2}+\sum {\left[A(f^{\prime })\right]}^{2}}\times 100\%$$

where f and $f^{\prime }$ denote the fault component and noise component, respectively, A(f) and $A(f^{\prime })$ denote their amplitudes in the frequency spectrum, respectively. The denominator part is the sum of the envelope spectral amplitude for the time domain signal, and the numerator part is the sum of the envelope spectral amplitude for the fault characteristic frequency and its multiples.

Table 4 shows the FFC values of the four methods, and the results demonstrate the superiority of the proposed method in this study. Table 5 shows the average CPU times of the four methods with 10 tests.

## 6. Conclusions

A compound fault diagnosis method for wind turbine gearboxes based on MOMEDA and the parallel parameter optimized RSSD is proposed in this study. MOMEDA obtains the deconvolution period based on the fault frequency and obtains periodic continuous pulses in a non-iterative deconvolution manner, thus decoupling and separating the compound fault vibration signals. However, some weak fault features are easily buried in transmission channels and background noise and using MOMEDA alone is not immune to dealing with multiple faults. Therefore, its combination with RSSD for parallel parameter optimization is applied to suppress disturbances and enhance the relevant fault characteristics. The parallel parameter optimized RSSD takes the composite indicator with low resonance components as the objective function and adaptively obtains the best quality factor Q and redundancy r according to the signal properties. The composite indicator measures the periodicity of the signal while measuring impulsivity, thus improving the signal sparse representation performance of RSSD. A bank of base functions matching the fault characteristics with the selected optimal parameters was constructed. RSSD adaptively decomposes the fault signal into high-resonance components and low-resonance components. The feasibility of the proposed method is verified by actual fault signals of wind turbine gearboxes. Compared with MCKD and MCKD-RSSD methods, the proposed method not only possesses excellent performance of decoupling separation and enhancing weak fault characteristics but also clearly and accurately portrays the time-frequency domain characteristics of different fault types, which is more suitable for composite fault diagnosis of wind turbine gearboxes. However, the method also has some drawbacks, such as time-consuming computation time during parameter optimization. In further studies, we will improve this problem to reduce the computation time.

## Figures and Tables

**Table 1:** Parallel shaft gearbox parameters.

Gear Element	No. of Teeth	Root Diameter (mm)	Helix Angle	Face Width (mm)
Intermediate gear	82	678	14R	170
Intermediate pinion	23	174	14L	186
High-speed gear	88	440	14R	110
High-speed pinion	22	100	14L	120

**Table 2:** High-speed shaft bearing parameters.

Bearing Pitch diameter/mm	Large End of Rolling diameter/mm	Small End of Rolling diameter/mm	Number of rollers/N	Contact Angle (*α*/degree)
155.00	24.22	19.03	20	11.63

**Table 3:** Characteristic frequency of faulty parts.

HSS Pinion Frequency $f_{r1}$	IMS Gear Frequency $f_{r2}$	Meshing Frequency $f_{m}$	Bearing Inner Ring Fault $f_{i}$	Bearing Rollers Fault $f_{b}$
30.00	7.50	660.00	345.30	93.51

**Table 4:** Performance indexes of the method in this paper and the comparative methods.

Value	MCKD	MCKD + RSSD	MOMEDA	Proposed Method
HSS pinion fault for FFC	9.34%	15.55%	11.10%	29.76%
IMS gear fault for FFC	27.55%	48.12%	56.24%	65.47%
Bearing inner ring fault for FFC	0.68%	1.35%	1.64%	5.83%
Bearing rollers fault for FFC	1.20%	4.17%	3.40%	11.83%

**Table 5:** Comparison of the average running time of different methods.

Method	MCKD	MCKD + RSSD	MOMEDA	Proposed Method
Time(s)	23.69	450.48	4.58	280.85

## Data Availability

Not applicable.
